# Validation of the IPF-specific version of St. George’s Respiratory Questionnaire

**DOI:** 10.1186/s12931-019-1169-9

**Published:** 2019-08-28

**Authors:** Thomas Skovhus Prior, Nils Hoyer, Saher Burhan Shaker, Jesper Rømhild Davidsen, Janelle Yorke, Ole Hilberg, Elisabeth Bendstrup

**Affiliations:** 1 Department of Respiratory Diseases and Allergy, Aarhus University Hospital, Aarhus, Denmark; 2 Department of Respiratory Medicine, Herlev-Gentofte University Hospital, Copenhagen, Denmark; 3 Department of Respiratory Medicine, Odense University Hospital, Odense, Denmark; 4 University of Manchester and The Christie NHS Foundation Trust, Manchester, UK; 5 Department of Respiratory Medicine, Vejle Hospital, Vejle, Denmark

**Keywords:** Interstitial lung disease, Idiopathic pulmonary fibrosis, Health-related quality of life, Quality of life, IPF-specific version of the St. George’s respiratory questionnaire, SGRQ-I, SGRQ, K-BILD

## Abstract

**Background:**

Patients with idiopathic pulmonary fibrosis (IPF) have impaired health-related quality of life (HRQL). To measure HRQL, an IPF-specific version of the St. George’s Respiratory Questionnaire (SGRQ-I) was developed, but not sufficiently validated. This study aimed to assess the validity (i.a. known-groups validity and concurrent validity) and test-retest reliability of SGRQ-I in IPF patients with different disease durations.

**Methods:**

Patients with IPF were consecutively recruited and completed SGRQ, SGRQ-I, King’s Brief Interstitial Lung Disease questionnaire (K-BILD), University of California, San Diego Shortness of Breath Questionnaire (SOBQ) and Short Form-36 (SF-36) along with pulmonary function tests and a 6-min walk test (6MWT) at baseline. After two weeks, SGRQ-I and Global Rating of Change Scales (GRCS) were completed.

**Results:**

At baseline and after two weeks, 150 and 134 patients completed the questionnaires, respectively. The internal consistency of SGRQ-I was high (Cronbach’s α = 0.92). Good concurrent validity was demonstrated by high intraclass correlation coefficients (ICC = 0.97), Bland-Altman plots and moderate to strong correlations to K-BILD, SOBQ and SF-36 (r = − 0.46 to 0.80). High ICC (0.92) and a Bland-Altman plot indicated good test-retest reliability. SGRQ-I was good at discriminating between patients with different stages of disease (Δscore > 18.1, effect sizes > 0.10). Validity was similar across groups of different disease duration.

**Conclusions:**

SGRQ-I proved to be valid at distinguishing between different disease severities, valid compared to other HRQL instruments, applicable across different disease durations and reliable upon repetition. SGRQ-I is a valid option for measuring HRQL in patients with IPF.

**Trial registration:**

The study was registered at clinicaltrials.org (NCT02818712) on 15 June 2016.

**Electronic supplementary material:**

The online version of this article (10.1186/s12931-019-1169-9) contains supplementary material, which is available to authorized users.

## Background

Idiopathic pulmonary fibrosis (IPF) is a progressive interstitial lung disease (ILD) with a poor prognosis [1]. Patients with IPF experience both physical and psychological deficits including dyspnea, reduced exercise capacity, social isolation and loss of mental well-being [2]. These symptoms inevitably affect the quality of life of patients with IPF.

Health-related quality of life (HRQL) expresses of the impact of a patient’s health status on his or her quality of life. As the current treatments for IPF do not significantly reduce mortality [3, 4], improving HRQL is becoming an important outcome in both clinical trials and daily clinical practice. HRQL can be measured using both generic and disease-specific instruments [5]. Disease-specific instruments have been designed to assess aspects of health status particularly relevant to the disease of interest. This improves the relevance of the items of the instrument to patients and will probably make them more responsive to changes than generic instruments [5].

Often, non-IPF specific instruments have been used to assess HRQL in patients with IPF e.g. the St. George’s Respiratory Questionnaire (SGRQ) [3, 6]. SGRQ was originally developed for patients with obstructive lung diseases [7, 8], but due to a lack of disease-specific HRQL instruments, SGRQ has been widely used in patients with IPF. Even though SGRQ holds acceptable validity and reliability in patients with IPF, some items are less relevant to this patient group and possesses weaker psychometric properties [7]. Among these, especially the symptoms domain including questions about attacks of chest trouble and wheezing are less relevant to patients with IPF.

An IPF-specific version of the SGRQ (SGRQ-I) was developed based on a cohort of patients with IPF [9]. Of the 50 items in SGRQ, the 34 items which were most reliable for measuring HRQL in patients with IPF were retained in SGRQ-I. However, important aspects of validity have not been assessed in SGRQ-I. To our knowledge, no previous studies have examined the ability of SGRQ-I to distinguish between patients with different stages of disease severity. This is a substantial part of validity, as the instrument should be able to discriminate patients with advanced disease from patients in early disease states. Neither has SGRQ-I been compared to a dyspnea instrument which is validated for use in patients with IPF nor to another ILD-specific HRQL instrument. A number of instruments are used to measure dyspnea, but the University of California, San Diego Shortness of Breath Questionnaire (SOBQ) is one of the best validated instruments for use in patients with IPF [10, 11]. The King’s Brief Interstitial Lung Disease questionnaire (K-BILD) is an ILD-specific instrument measuring HRQL that has high validity in patients with IPF [12]. By comparing SGRQ-I to such instruments, the validity of the questionnaire can be strengthened. Furthermore, test-retest reliability of SGRQ-I has only been examined in a small study of 23 patients with IPF [13]. It is essential that the results of the instrument are repeatable with minimal variation in stable patients.

To increase the generalizability and reliability of SGRQ-I, the results of the initial validation should be repeatable in other cohorts of patients with IPF. Also, the validity should be examined in both patients with a recent diagnosis of IPF and longer disease durations. Another aspect of generalizability is the use of instruments in other languages. So far, SGRQ-I has only been translated into Spanish [13], and no IPF-specific HRQL instruments are available in Danish. Translation of valid and reliable HRQL instruments is important to support international research in new IPF treatments and studies aiming at uncovering determinants of HRQL in patients with IPF. This is needed to make effective interventions targeted at improving HRQL in patients living with this burdensome disease. Thus, efforts might include discussing advance care planning and palliation at an early stage in patients with this progressive disease, which is also recommended by the World Health Organization (WHO) [14, 15].

The aim of this study was to evaluate the known-groups validity and test-retest reliability of SGRQ-I, assess the validity of SGRQ-I in patients with different disease durations, translate SGRQ-I into Danish and examine the correlations to SOBQ and K-BILD.

## Methods

### Translation and cultural adaptation

Minor parts of SGRQ were changed during the development of SGRQ-I [9]. These passages were translated into Danish by a stepwise forward-backward translation procedure (see Additional file 1) [16, 17]. Semi-structured interviews with a group of patients with IPF were performed to obtain the patients’ perspective on the translated version of SGRQ-I. The modified versions of SGRQ-I were reviewed by the developers during the course of translation and at the final approval. The SGRQ-I was composed of the translated passages and the existing translation of SGRQ.

### Design

Patients diagnosed with IPF were recruited successively from the three tertiary ILD centres in Denmark at the University Hospitals in Aarhus, Gentofte (Copenhagen) and Odense. Adult patients with a guideline-based diagnosis of IPF were eligible for inclusion [18, 19]. Both prevalent and incident patients were included. The only exclusion criterion was inability to complete the questionnaires due to cognitive or linguistic barriers. The same patient cohort was also used to validate the K-BILD (manuscript submitted).

At baseline, patients completed SGRQ-I, SGRQ, Short Form-36 (SF-36) and SOBQ. Fourteen days later, SGRQ-I and Global Rating of Change Scales (GRCS) were completed. Questionnaires missing total or domain scores or containing more than 15% missing answers were excluded from the analyses. Results were obtained from pulmonary function tests (PFTs)(forced vital capacity (FVC) and diffusing capacity of the lung for carbon monoxide (DLCO)), and the 6-min walk test (6MWT), and the gender, age and physiology (GAP) index was calculated [20].

The study was approved by the Danish Data Protection Agency and the Central Denmark Region Committee of Health Research Ethics. The study was registered at clinicaltrials.org (https://clinicaltrials.gov/ct2/show/NCT02818712). Written and informed consent was obtained from participants before enrolment in the study.

### Study measures

*SGRQ-I* consists of 34 self-completed items measuring HRQL [9]. It was developed as an IPF-specific version of SGRQ. Different scales are used to score SGRQ-I and results in a total score and three domain scores: Impacts, Activities and Symptoms. Scores range from 0 to 100, with higher scores indicating more impaired HRQL.

*SGRQ* is a 50-item self-completed questionnaire assessing HRQL [8]. It was developed for patients with chronic obstructive lung disease (COPD) and asthma, but has subsequently been validated for patients with IPF [7]. Response options, scoring and domains are similar to SGRQ-I. Higher scores also correspond to more impaired HRQL.

*K-BILD* consists of 15 self-completed items assessing HRQL in patients with ILD [12]. Answers are scored on a 7-point Likert scale and results in a total score and three domain scores: Chest symptoms, Breathlessness and activities and Psychological. Scores range from 0 to 100, with higher scores corresponding to better HRQL.

*SOBQ* estimates dyspnea associated with activities of daily living in a 24-item self-completed questionnaire [21]. Symptoms are scored on a 6-point scale. Scores range from 0 to 120 and higher scores denote more dyspnea.

*SF-36* contains 36 self-completed items concerning generic quality of life and is scored on a 3–6-point Likert scale [22]. Scoring is divided into eight domain scores and two component scores, based on scores ranging from 0 to 100. High quality of life is expressed by high scores.

*GRCS* are designed to assess the current state of the patients compared to baseline [23]. The questionnaires are self-completed on an 11-point Likert scale. Responses range from “Very much worse” over “Unchanged” to “Very much better” with corresponding numbers ranging from − 5 to 5. Four GRCS scores composed: One for overall health status and three for the SGRQ-I domains.

*The GAP index* is a prognostic staging system developed to predict mortality in patients with IPF [20]. The index is a composite score, which is calculated based on gender, age and 2 lung physiology variables (FVC and DLCO). The patients are divided into three groups with different 1-year mortalities ranging from 6 to 39%.

### Validation

The interrelatedness of the items in SGRQ-I was examined to measure the *internal consistency* of the questionnaire. *Concurrent validity* was evaluated by comparing SGRQ-I to SGRQ, K-BILD, SOBQ, SF-36, PFTs and distance walked during the 6-min walk test (6MWD). *Test-retest reliability* was evaluated by comparing the SGRQ-I scores at baseline and after two weeks in stable patients. *Known-groups validity* was evaluated by estimating the ability of the SGRQ-I to distinguish groups of patients with different stages of disease severity. Stratification of patients into “known groups” of disease severity was performed in accordance with their PFTs (FVC and DLCO divided into quartiles), supplement of long-term oxygen therapy (LTOT) and their GAP index [24].

### Statistical analysis

Patients were categorized into three groups in accordance with the time since diagnosis of IPF (TSD): < 1 month, 1–12 months and > 12 months. The thresholds were chosen to reflect newly diagnosed patients, patients with a short disease duration and, lastly, patients with a longer disease duration.

Fisher’s exact test for binomial data was used to compare the characteristics of non-responders vs. responders of the questionnaires at baseline and after two weeks. Cronbach’s α was calculated for each domain and total score to assess the internal consistency of SGRQ-I. Results above 0.7 are regarded as reliable internal consistency [25].

Concurrent validity was examined by intraclass correlation coefficients (ICCs) and Bland-Altman plots for comparison of SGRQ-I and SGRQ, and Pearson’s correlation coefficients were used to compare SGRQ-I to the other measures after evaluation of linearity. Test-retest reliability was also evaluated by ICCs and Bland-Altman plots after assessment of normality. Patients scoring − 1 to 1 in GRCS two weeks after baseline were considered stable. ICC values above 0.7 are accepted as valid measures of equivalence and reliability [25].

Continuous data were analysed by the independent two-sample t-test when normally distributed, and by the Wilcoxon-Mann-Whitney test when not normally distributed. Linear regression analysis was applied for comparison of GAP groups, and the model was checked by diagnostic plots of the residuals. Analysis of variance (ANOVA) or multiple linear regression was performed to subsequently calculate effect size, reported as partial η^2^: 0.01 = small effect, 0.06 = medium effect and 0.14 = large effect [26]. Bartlett’s test for equal variances and diagnostic plots of the residuals were used for model checking of ANOVA.

Quantile-quantile plots (QQ-plots) were used to assess normality, and the F-test was used to assess variance homogeneity. Data were analysed using STATA, version 14.2.

## Results

### Translation and cultural adaptation

The permission to translate SGRQ-I was obtained from the developers of the original instrument [9]. The Danish version of SGRQ-I was accepted with a minor revision by the developers, after the forward-backward translation procedure. Semi-structured interviews of a representative group of five patients with IPF (see Additional file 2) were performed after completing the Danish version of SGRQ-I. The patients thought the SGRQ-I was comprehensive with a high face and content validity. No changes were made to the Danish version after the interviews (see Additional file 3).

### Psychometric validation

Between August 2016 and March 2018, 150 patients with IPF were included in the study from the three tertiary ILD centers in Denmark (110 patients in Aarhus, 24 in Gentofte and 16 in Odense). Demographics of the patients are presented in Table 1.

**Table 1:** Baseline demographics of participants (*n* = 150)

Characteristics	Value
Male (%)	122 (81.3%)
Age, years ± SD	72.9 ± 6.2
Smoking status	
Never (%)	40 (26.6%)
Former (%)	101 (67.3%)
Current (%)	9 (6.0%)
Time since diagnosis, years (interquartile range)	0.5 (0.0–9.2)
< 1 month	0.0 (0.0–0.02)
1–12 months	0.5 (0.3–0.8)
> 12 months	2.8 (1.8–4.0)
FVC, % predicted ± SD	87.2 ± 23.1
DLCO, % predicted ± SD	48.4 ± 14.1
6MWD, m ± SD	450.3 ± 112.5
Long-term oxygen therapy (%)	19 (12.7%)

Values are presented as *n* (%), mean ± standard deviation (SD) or mean with interquartile range. *FVC*: Forced vital capacity; *DLCO*: Diffusing capacity of the lung for carbon monoxide; *6MWD*: distance walked during the 6-min walk test. The same patients were used in a validation study of K-BILD (manuscript submitted)

The number of questionnaires missing domain or total scores or comprising more than 15% missing answers at baseline were: SGRQ-I (2 missing, 1.3%), K-BILD (1 missing, 0.7%), SOBQ (3 missing, 2.0%), SGRQ (2 missing, 1.3%) and SF-36 (1 missing, 0.7%). Only one item had a substantial number of missing answers (I1, 49,3% missing) (see Additional file 4). After two weeks, nine patients had more than 15% missing answers and seven patients did not complete the questionnaires (4.7%). Missing data analyses revealed no differences between responders and non-responders at baseline (see Additional file 5). After two weeks, statistically significant differences were weak and only included smoking status (*p* = 0.03) and 6MWD (*p* = 0.04). Responders after two weeks walked on average 62.7 m longer than non-responders during the 6MWT. There were more former smokers among the responders and more current and never smokers among the non-responders (see Additional file 5). No floor or ceiling effects were observed in neither SGRQ-I total nor domain scores, as < 15% of the patients obtained the highest or lowest possible scores, respectively [27].

### Internal consistency

Cronbach’s α was high in both total and domain scores of SGRQ-I indicating a good internal consistency (Table 2). The results were comparable in the TSD subgroups (data not shown), except for the symptoms domain having a slightly smaller Cronbach’s alpha value of 0.67.

**Table 2:** Internal consistency and concurrent validity of SGRQ-I

SGRQ-I	Cronbach’s α	ICC
Total	0.92	0.97
Symptoms	0.77	0.78
Activities	0.86	0.94
Impacts	0.84	0.96

Data are presented as Cronbach’s alpha and intraclass correlation coefficients (ICC) of the total and three domain scores of SGRQ-I compared to SGRQ for all patients. *SGRQ-I*: IPF-specific version of the St. George’s Respiratory Questionnaire

### Concurrent validity

**Fig. 1:**
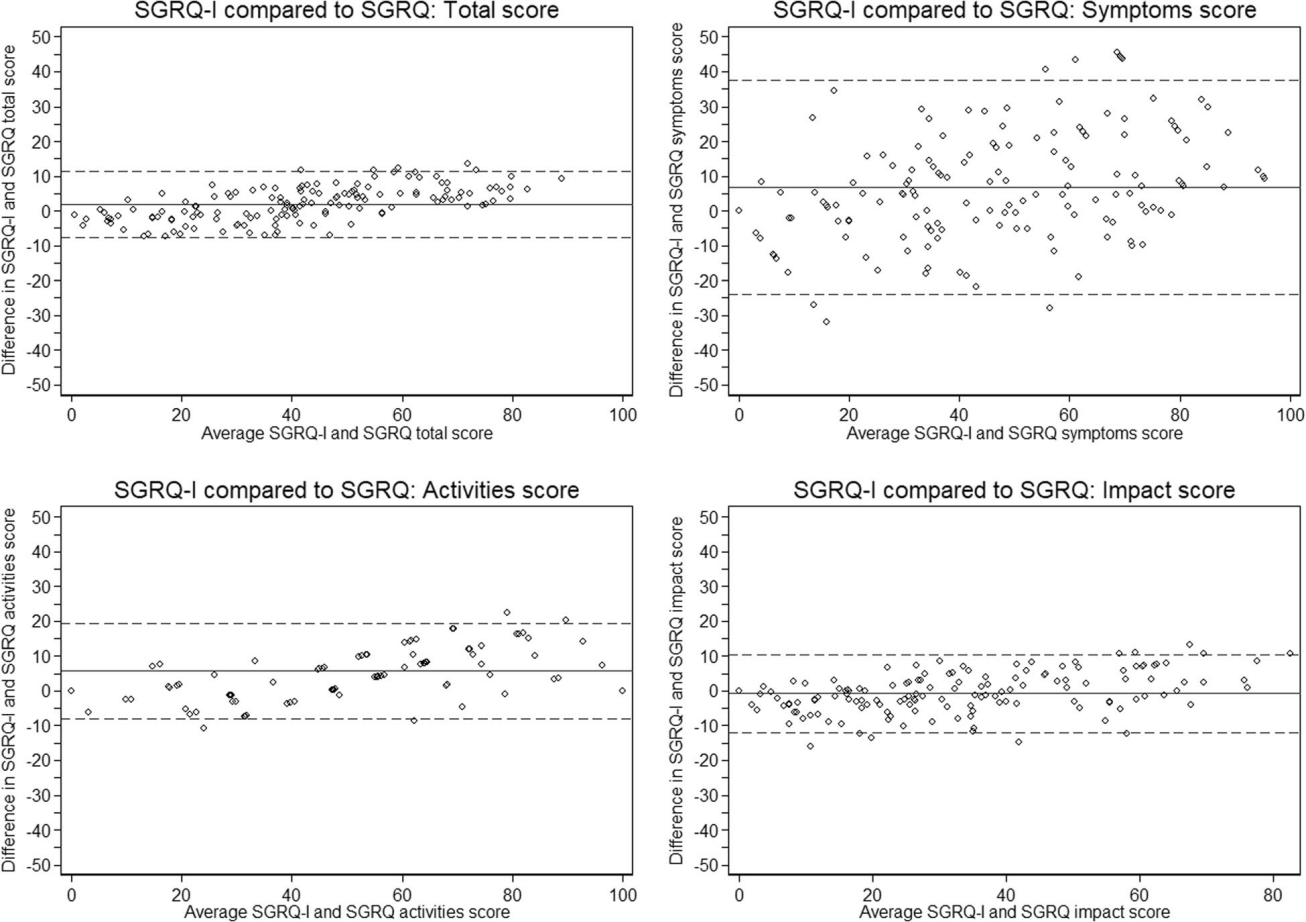
Bland-Altman plot of the agreement between SGRQ-I and SGRQ for all patients. The solid line is the mean difference, while the dashed lines are the 95% limits of agreement. *SGRQ-I*: IPF-specific version of the St. George’s Respiratory Questionnaire; *SGRQ*: St. George’s Respiratory Questionnaire

Agreement between SGRQ-I and SGRQ measured by ICCs was good in the symptoms domain and excellent in the total and other domain scores (Table 2). Bland-Altman plots supported these findings, even though SGRQ-I tended to score a bit higher in the symptoms and activities domains and there was a slight incline in the difference between the two scores with increasing average scores (Fig. 1). Correlations to K-BILD, SOBQ and SF-36 were mainly moderate to strong, while correlations were weaker to PFTs and 6MWD (Table 3). Overall, these findings indicate a good concurrent validity.

**Table 3:** Concurrent validity of SGRQ-I

	SGRQ-I Total	SGRQ-I Symptoms	SGRQ-I Activities	SGRQ-I Impacts
K-BILD total	−0.76*	− 0.58*	− 0.71*	−0.70*
K-BILD chest symptoms	−0.69*	−0.64*	− 0.54*	−0.66*
K-BILD breathlessness and activities	−0.78*	−0.57*	− 0.76*	−0.70*
K-BILD psychological	−0.58*	−0.47*	− 0.52*	−0.55*
SOBQ total	0.80*	0.54*	0.74*	0.76*
SF-36 PCS	−0.71*	− 0.50*	−0.63*	− 0.68*
SF-36 MCS	−0.46*	− 0.40*	−0.34*	− 0.46*
FVC%	−0.30*	− 0.32*	−0.20*	− 0.30*
DLCO%	−0.48*	− 0.28*	−0.53*	− 0.42*
6MWD (m)	−0.50*	− 0.25*	−0.46*	− 0.52*

All data are presented as Pearson’s correlation coefficients for all patients. *: *p* < 0.02. *SGRQ-I*: IPF-specific version of the St. George’s Respiratory Questionnaire; *K-BILD*: King’s Brief Interstitial Lung Disease questionnaire; *SOBQ*: University of California, San Diego Shortness of Breath Questionnaire; *SF-36*: Short Form-36; *PCS*: Physical Component Score; *MCS*: Mental Component Score; *FVC*: Forced vital capacity; *DLCO*: Diffusing capacity of the lung for carbon monoxide; *6MWD*: Distance walked during the 6-min walk test

The three TSD subgroups had similar ICCs and correlations, apart from the 6MWD and DLCO. Correlation between DLCO and the activities domain was weaker for patients with an IPF diagnosis < 1 month (− 0.21). 6MWD had weaker correlations to the activities and impacts domains in patients with an IPF diagnosis < 1 month (− 0.04 and − 0.26, respectively). Overall, these findings indicate a good concurrent validity.

### Test-retest reliability

**Fig. 2:**
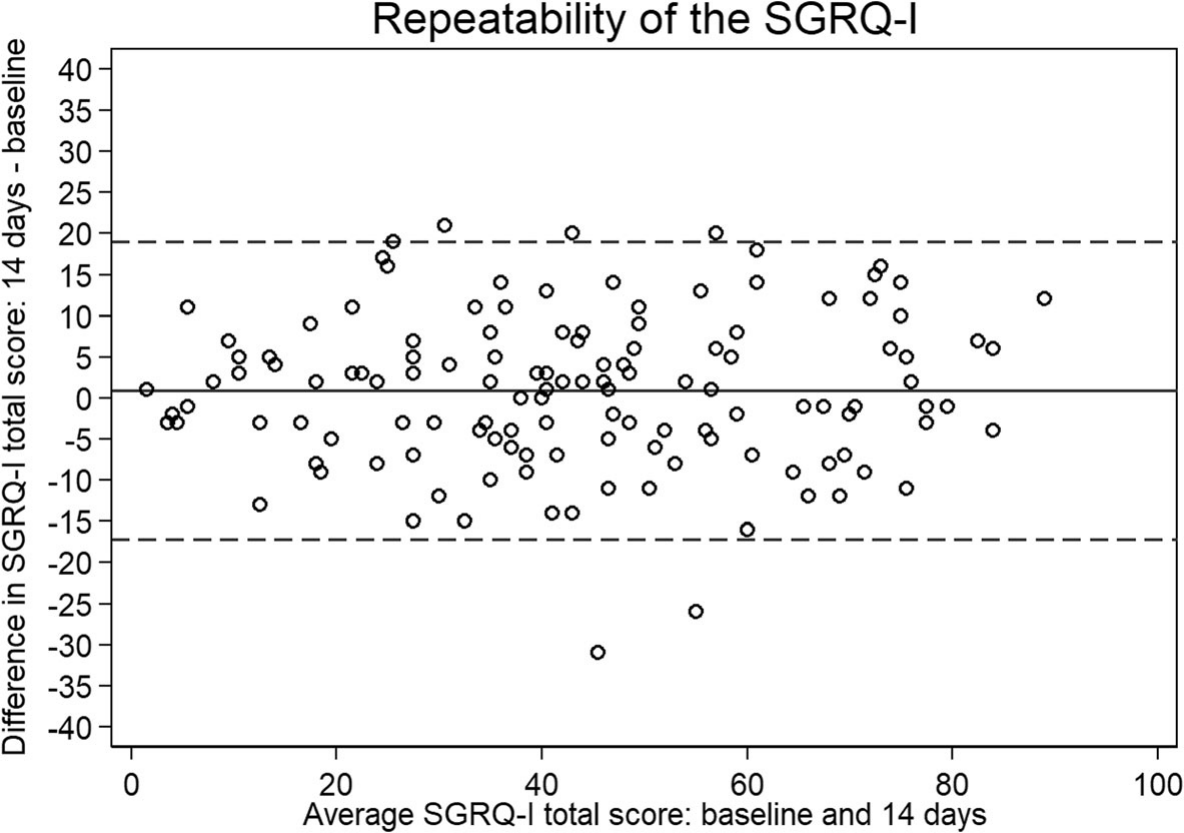
Bland-Altman plot of the repeatability of SGRQ-I in all stable patients. The solid line is the mean difference, while the dashed lines are the 95% limits of agreement. *SGRQ-I*: IPF-specific version of the St. George’s Respiratory Questionnaire

Most patients were stable in overall health status as well as in the three domains of SGRQ-I, as evaluated by GRCS after two weeks. A good test-retest reliability of SGRQ-I was seen in the stable patients by high ICC values and a Bland-Altman plot when comparing answers at baseline and after two weeks (Table 4 and Fig. 2). The results were comparable across the TSD groups (data not shown).

**Table 4:** Test-retest reliability of SGRQ-I

SGRQ-I	*n*	ICC
Total	99 (73.9%)	0.92
Symptoms	105 (78.4%)	0.81
Activities	104 (77.6%)	0.80
Impacts	104 (77.6%)	0.79

Data represent number of stable patients (% of responders, *n* = 134) and intraclass correlation coefficients (ICCs). *SGRQ-I*: IPF-specific version of the St. George’s Respiratory Questionnaire

### Known-groups validity

**Fig. 3:**
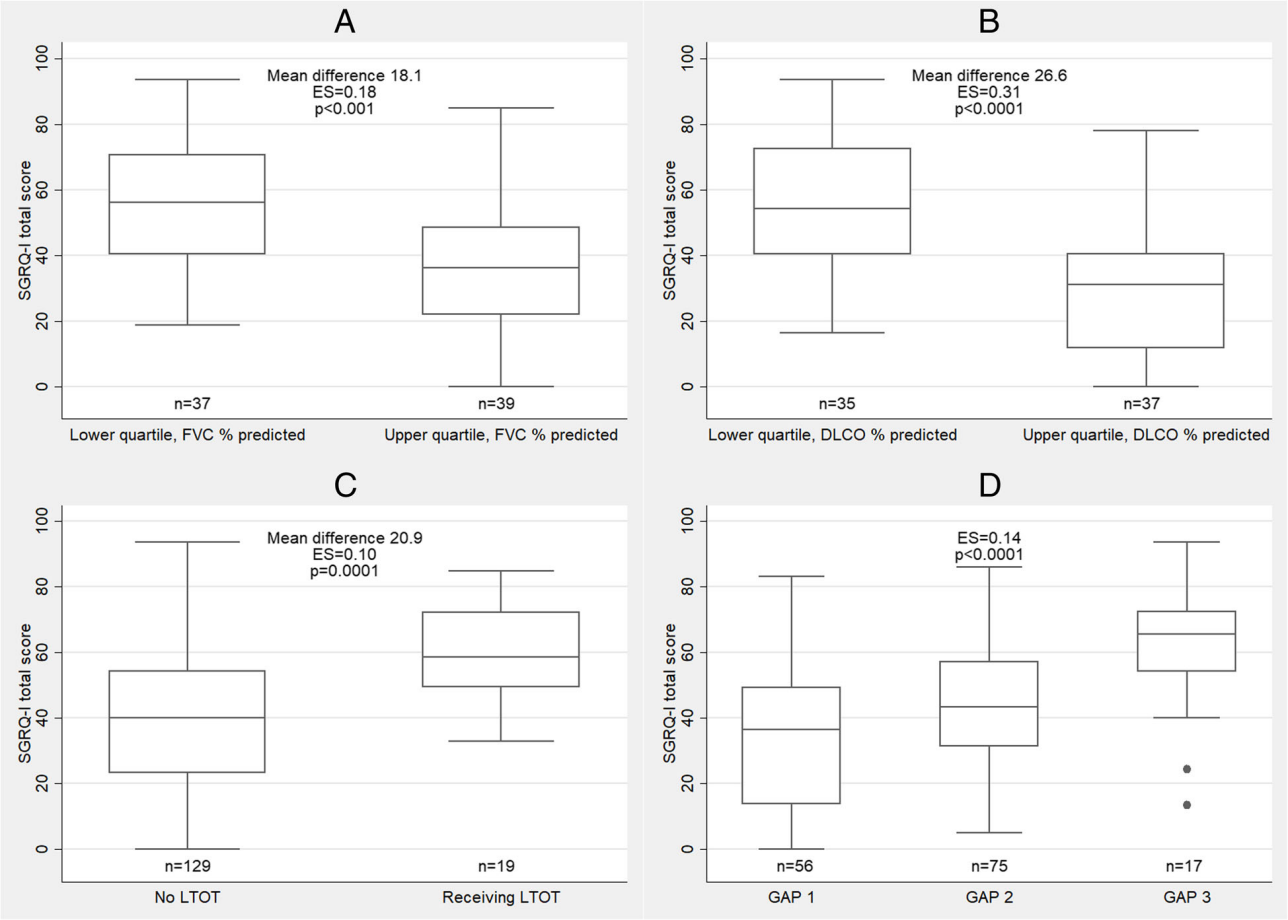
SGRQ-I total score in (**a**) the lower and upper quartile of FVC % predicted, (**b**) the lower and upper quartile of DLCO % predicted, (**c**) long-term oxygen therapy and (**d**) GAP index. The midlines in the boxes are the median values and the boundaries are the 25th and 75th percentiles; the whiskers are the upper adjacent values (1.5 interquartile range above the 75th percentile) and lower adjacent values (1.5 interquartile range below the 25th percentile). The dots are outlying values. *SGRQ-I*: IPF-specific version of the St. George’s Respiratory Questionnaire. *FVC*: Forced vital capacity; *DLCO*: Diffusing capacity of the lung for carbon monoxide; *LTOT*: Long-term oxygen therapy; *GAP*: Gender, age, physiology

SGRQ-I total scores were significantly higher in patients in the lower quartile of FVC % predicted and DLCO % predicted compared to patients in the upper quartile (Fig. 3 and Additional file 6). Patients receiving LTOT had significantly higher SGRQ-I total scores than patients not receiving oxygen therapy. SGRQ-I total score increased with advancing disease severity reflected by the GAP index. Medium to large effect sizes supports the high discriminative strength of SGRQ-I.

## Discussion

SGRQ-I was translated into Danish and proved to be a valid tool to measure HRQL with a good internal consistency, solid concurrent validity, high test-retest reliability and a good ability to discriminate between patients with different stages of disease. SGRQ-I was also equally valid in patients with different disease durations on almost all parameters.

The known-groups validity of SGRQ-I has not previously been investigated. An important aspect of measurement validity is the ability of the instrument to distinguish between patients with different stages of disease, as HRQL worsens with increasing disease severity [28]. Our results show that SGRQ-I is very good at differentiating patients with respect to pulmonary function measured by FVC and DLCO. When stratifying patients into groups according to the GAP index or use of LTOT, SGRQ-I was also able to differentiate between these groups. These novel results add further weight to the validity of SGRQ-I and emphasizes the relevant utility of the instrument.

Reliability was not assessed during the development of the instrument and was only evaluated in a small group of 23 patients in another study. Reliability is a central part of an instrument’s measurement qualities to supply trustworthy results. SGRQ-I proved to be very reliable when completed twice within a short period of time in stable patients. Apart from the limited sample size, patients were only asked for worsening of symptoms upon completing SGRQ-I the second time [13]. We excluded patients with both improvement and deterioration to ensure that only truly stable patients were included in the analysis of reliability.

In order to examine the concurrent validity, we compared SGRQ-I to SGRQ and correlated SGRQ-I to other HRQL instruments and measurements of disease severity relevant to IPF. The ICCs were high for both domain and total scores, indicating very good agreement between SGRQ-I and SGRQ. The Bland-Altman plots supported these findings, even though there was a tendency towards slightly higher scores in SGRQ-I compared to SGRQ with increasing average scores. As such, SGRQ-I scores indicate a broader spectrum of HRQL, as patient have better HRQL measured by SGRQ-I than by SGRQ with low average scores and worse HRQL with higher average scores. This may be due to the removal of selected item with poor fit to the Rasch model or many missing answers in patients with IPF [9]. If the two instruments had very similar results, the justification for SGRQ-I would only lie in face and content validity. Based on these results, one could argue that SGRQ-I should be used instead of SGRQ in patients with IPF, as the results differ slightly and SGRQ-I is targeted at IPF. The validity of SGRQ-I is also supported by the strong correlations to K-BILD. After all, comparing SGRQ-I to an ILD-specific HRQL instrument provides better evidence of the validity than comparisons to instruments developed for other lung diseases.

Compared to SGRQ, the SGRQ-I holds a pronounced advantage as it only consists of 34 items compared to 50 items in the SGRQ. It is easier to complete and has the same validity and reliability as SGRQ. Nevertheless, both instruments are more suitable for research purposes than clinical assessments. A Tool to Assess Quality of life in IPF (ATAQ-IPF) is another IPF-specific HRQL instrument containing 74 items [29]. ATAQ-IPF covers more domains than SGRQ-I but is also more time consuming to complete which may limit its use. As such, SGRQ-I should be considered as an IPF-specific HRQL instrument in future clinical trials. Other HRQL questionnaires validated for IPF and other ILDs include K-BILD and the COPD Assessment Test (CAT). K-BILD consists of 15 items and has validity and reliability comparable to SGRQ-I [12]. CAT was developed for patients with COPD, but has subsequently been validated in IPF and other ILDs [30–32]. However, as SGRQ-I is more comprehensive than both K-BILD and CAT, doctors and healthcare professionals will have a better impression of the disabilities and limitations experienced by the patients in their daily living. Hence, it will be easier to intervene and assist the patients in an attempt to improve their everyday HRQL.

Dyspnea is a major symptom in IPF and correlations to SOBQ were generally strong, demonstrating a good reflection of this symptom in SGRQ-I. In the original version, dyspnea was measured using the Borg dyspnea index and the baseline dyspnea index (BDI). The correlation of SGRQ-I total score to SOBQ was stronger than the correlations to Borg scale and BDI (0.80 vs 0.46 and − 0.67, respectively). SOBQ has been validated for use in patients with IPF [10, 11] and covers dyspnea associated with a wide range of daily activities. As such, SOBQ may be a better measure of dyspnea in IPF than Borg and BDI, and SGRQ-I seem to capture the severity of dyspnea very well.

Correlations to the generic SF-36 confirmed the concurrent validity of SGRQ-I, although the correlations were mainly weaker than correlations to the other HRQL instruments. This is probably caused by the generic nature of the SF-36, which has to be applicable across a wide range of conditions and is not tailored to reflect the symptoms and implications of living with for instance IPF in the same way as disease-specific HRQL instruments. The mental component score had weaker correlations than the physical component score. Comparable result were obtained in the initial development and validation of SGRQ-I [9]. As the psychological domain of K-BILD also had weaker correlations to SGRQ-I, the psychological impact of living with IPF may be more diffuse and difficult to incorporate into a HRQL instrument than the physical symptoms accompanying IPF.

Correlations to FVC were weaker than correlations to DLCO and 6MWD. Even though SGRQ-I had moderate correlations to PFT results and 6MWD, these measures only estimate the physiological limitations of IPF and not the full impact of IPF on the patients’ lives. Similarly, moderate to weak correlations have been demonstrated in other HRQL questionnaires including SGRQ, K-BILD and ATAQ-IPF [7, 12, 29]. Therefore, HRQL instruments are important supplements for both clinical trials and daily clinical practice to get a full picture of the current state of patients with IPF.

This study included the largest number of patients in a translation and validation study of SGRQ-I, which has previously only been translated into Spanish in a population of only 23 patients [13]. By including a larger cohort of patients, the generalizability of our results increases, as the study population is more likely to reflect the background population in terms of disease severity, socio-economic status and views on life. Our results support the former findings indicating that SGRQ-I is a valid and reliable measure of HRQL [9, 13]. Also, SGRQ-I proved to be equally valid in patients with different disease durations which is a novel finding. The weaker correlations of the activities and impacts domains to DLCO and 6MWD in incident patients do not significantly change these results.

SGRQ-I is currently the only tool in Danish to measure HRQL explicitly developed for patients with IPF. The questionnaire was both well-received and perceived as relevant by patients with IPF. The Danish version of SGRQ-I was comparable to the original English version and as such, SGRQ-I performed well in a non-English speaking population.

Responders and non-responders were comparable regarding demographics, LTOT, medical treatment or PFTs in the missing data analyses at baseline. After two weeks, the only significant differences were smoking status and 6MWD. Though these results could indicate some degree of healthy volunteer bias, however, as differences between the two groups were minimal, we presume that no significant selection bias was introduced.

The large number of participants is a clear strength of our study. Also, the fact that the patients were recruited in a multicenter setup increased the generalizability of the results with a better reflection of the background IPF population. Furthermore, we assessed many different aspects of validity and reliability, including comparisons to both other HRQL instruments and measures of disease severity. A limitation of our study is the single measurement of pulmonary function and level of physical activity. Symptoms can vary from day to day, and repeated measurements at home, e.g. with home spirometry or accelerometers, might give a better impression of the true physical functional state of the patients.

## Conclusions

SGRQ-I is a valid measure of HRQL in patients with IPF that can be utilized in patients with different disease durations. SGRQ-I can discriminate between patients with different stages of disease severity and is reliable upon repeated measurements in stable patients. The impact of dyspnea on HRQL is well represented in SGRQ-I and HRQL measured by SGRQ-I reflect the results of another ILD-specific HRQL instrument. Translation of SGRQ-I into another language with equal validity is feasible. Due to the poor prognosis of IPF and its progressive nature, HRQL is an important outcome in both daily clinical practice and clinical trials. As SGRQ-I is shorter than SGRQ but equally valid and reliable, SGRQ-I is an improvement and a better option for use in future clinical trials.

## Additional files

Additional file 1: Translation process. (DOCX 25 kb)

Additional file 2: Patients interviewed during the translation process. (DOCX 14 kb)

Additional file 3: Changes and comments in the translation process. (DOCX 14 kb)

Additional file 4: Missing items at baseline. (DOCX 15 kb)

Additional file 5: Missing data analyses. (DOCX 17 kb)

Additional file 6: Known groups validity. (DOCX 15 kb)

## Data Availability

The datasets collected and analyzed during the current study are not publicly available due to information that could compromise research participant privacy, but are available from the corresponding author on reasonable request.

## Abbreviations

6MWD: Distance walked during the 6-min walk test
ATAQ-IPF: A Tool to Assess Quality of life in IPF
CAT: The COPD Assessment Test
COPD: Chronic obstructive lung disease
DLCO: Diffusing capacity of the lung for carbon monoxide
FVC: Forced vital capacity
GAP: Gender, age and physiology
GRCS: Global Rating of Change Scales
HRQL: Health-related quality of life
ICC: Intraclass correlation coefficients
ILD: Interstitial lung disease
IPF: Idiopathic pulmonary fibrosis
K-BILD: The King’s Brief Interstitial Lung Disease questionnaire
LTOT: Long-term oxygen therapy
PFT: Pulmonary function test
QQ-plots: Quantile-quantile plots
SF-36: The Short Form-36 (SF-36)
SGRQ: The St. George’s Respiratory Questionnaire
SGRQ-I: The IPF-specific version of the St. George’s Respiratory Questionnaire
SOBQ: The University of California, San Diego Shortness of Breath Questionnaire
TSD: Time since diagnosis
WHO: World Health Organization
