# Supplementary material for: Validation of the IPF-specific version of St. George’s Respiratory Questionnaire
Source: Respir Res. 2019 Aug 28;20:199. doi: 10.1186/s12931-019-1169-9 (PMC6714302; doi:10.1186/s12931-019-1169-9)
Supplement: Supplementary file 1 — Translation process. (DOCX 25 kb) [file 12931_2019_1169_MOESM1_ESM.docx]

**Additional files**

**Additional file 1: Translation process**

*Q:* English version of SGRQ-I, *T:* Translation in Danish
