# Supplementary material for: Validation of the IPF-specific version of St. George’s Respiratory Questionnaire
Source: Respir Res. 2019 Aug 28;20:199. doi: 10.1186/s12931-019-1169-9 (PMC6714302; doi:10.1186/s12931-019-1169-9)
Supplement: Supplementary file 2 — Patients interviewed during the translation process. (DOCX 14 kb) [file 12931_2019_1169_MOESM2_ESM.docx]

**Additional file 2: Patients interviewed during the translation process**

| **Gender**  **(male/female)** | **Mean age**  **(range)** | **FVC % predicted (range)** | **DLCO % predicted**  **(range)** |
| --- | --- | --- | --- |
| 3/2 | 67 (53-72) | 82 (65-97) | 55 (42-61) |

*FVC*: Forced vital capacity; *DLCO*: diffusing capacity of the lung for carbon monoxide
