# Supplementary material for: Validation of the IPF-specific version of St. George’s Respiratory Questionnaire
Source: Respir Res. 2019 Aug 28;20:199. doi: 10.1186/s12931-019-1169-9 (PMC6714302; doi:10.1186/s12931-019-1169-9)
Supplement: Supplementary file 3 — Changes and comments in the translation process. (DOCX 14 kb) [file 12931_2019_1169_MOESM3_ESM.docx]

**Additional file 3: Changes and comments in the translation process**

**T1 -> T2 Question Changes**

Part 1, Heading “currently have” changed to “have at the moment”

Part 1, Q1 “I cough” changed to “I’m coughing”

Part 1, Q2 “I bring up phlegm (sputum)” changed to “I’m coughing up

mucus”

Part 1, Q3 “I have shortness of breath” changed to “I’m short of breath”

Part 1, Q4 “I have attacks of wheezing” changed to “I experience periods

of wheezing breath”

Part 1, Q5 “More than 1 attack” changed to “More than one bout”

Part 1, Q6 “None” changed to “Never”

Part 1, Q6 “A few days” changed to “Few days”

Part 2, Sect1 “My lung condition interferes or made me stop work” changed

to “My lung disease affects my work or has forced me to stop working”

Part 2, Sect1 “My lung problem does not affect my job” changed to “My

respiratory problems do not affect my work”

Part 2, Sect5 “which you think” changed to “which in your opinion”

**T2 -> T3 Question Changes**

Part 2, Sect1 “My respiratory problems do not affect my work” changed to

“My lung problems do not affect my work”

**T3 -> T4 No changes**

*T:* Translation in Danish, Q: Question, Sect: Section
