# Supplementary material for: Validation of the IPF-specific version of St. George’s Respiratory Questionnaire
Source: Respir Res. 2019 Aug 28;20:199. doi: 10.1186/s12931-019-1169-9 (PMC6714302; doi:10.1186/s12931-019-1169-9)
Supplement: Supplementary file 4 — Missing items at baseline. (DOCX 15 kb) [file 12931_2019_1169_MOESM4_ESM.docx]

**Additional file 4: Missing items at baseline**

| Item | Missing | Total | Percent Missing |
| --- | --- | --- | --- |
| S1 | 2 | 150 | 1.3 |
| S2 | 3 | 150 | 2.0 |
| S3 | 2 | 150 | 1.3 |
| S4 | 4 | 150 | 2.7 |
| S5 | 1 | 150 | 0.7 |
| S6 | 1 | 150 | 0.7 |
| A1 | 3 | 150 | 2.0 |
| A2 | 2 | 150 | 1.3 |
| A3 | 2 | 150 | 1.3 |
| A4 | 0 | 150 | 0.0 |
| A5 | 1 | 150 | 0.7 |
| A6 | 1 | 150 | 0.7 |
| A7 | 0 | 150 | 0.0 |
| A8 | 1 | 150 | 0.7 |
| A9 | 0 | 150 | 0.0 |
| A10 | 0 | 150 | 0.0 |
| I1 | 74 | 150 | 49.3 |
| I2 | 0 | 150 | 0.0 |
| I3 | 0 | 150 | 0.0 |
| I4 | 1 | 150 | 0.7 |
| I5 | 1 | 150 | 0.7 |
| I6 | 1 | 150 | 0.7 |
| I7 | 0 | 150 | 0.0 |
| I8 | 0 | 150 | 0.0 |
| I9 | 0 | 150 | 0.0 |
| I10 | 0 | 150 | 0.0 |
| I11 | 1 | 150 | 0.7 |
| I12 | 0 | 150 | 0.0 |
| I13 | 1 | 150 | 0.7 |
| I14 | 0 | 150 | 0.0 |
| I15 | 0 | 150 | 0.0 |
