# Supplementary material for: Validation of the IPF-specific version of St. George’s Respiratory Questionnaire
Source: Respir Res. 2019 Aug 28;20:199. doi: 10.1186/s12931-019-1169-9 (PMC6714302; doi:10.1186/s12931-019-1169-9)
Supplement: Supplementary file 5 — Missing data analyses. (DOCX 17 kb) [file 12931_2019_1169_MOESM5_ESM.docx]

**Additional file 5: Missing data analyses**

Baseline

|  | **Responders**  **(*n* = 145)** | **Non-responders**  **(*n* = 5)** | **Difference (95% CI)** | **p-value** |
| --- | --- | --- | --- | --- |
| **Gender** |  | | | |
| Male, *n* | 118 | 4 |  | 1.00 |
| Female, *n* | 27 | 1 |  |  |
| **Age,** years, mean ± SD | 72.9 ± 6.3 | 73.4 ± 3.4 | -0.5 (-6.1 to 5.1) | 0.86 |
| **Time since diagnosis**, years, median (range) | 0.5 (0.0 to 9.3) | 0.0 (0.0 to 3.3) |  | 0.26 |
| **Smoking status** |  | | | |
| Current, *n* | 8 | 2 |  | 0.12 |
| Former, *n* | 99 | 1 |  |  |
| Never, *n* | 38 | 2 |  |  |
| **Long-term oxygen therapy** |  | | | |
| No, *n* | 126 | 5 |  | 1.00 |
| Yes, *n* | 19 | 0 |  |  |
| **Medical treatment for IPF#** |  | | | |
| No, *n* | 62 | 3 |  | 0.65 |
| Yes, *n* | 83 | 2 |  |  |
| **FVC**, % predicted, mean ± SD | 87.2 ± 23.1 | 88.1 ± 23.2 | -1.0 (-21.8 to 19.8) | 0.93 |
| **DLCO**, % predicted, mean ± SD | 48.5 ± 14.2 | 47.5 ± 10.7 | 1.0 (-11.7 to 13.7) | 0.88 |
| **6MWD**, m, mean ± SD | 450.2 ± 113.5 | 455.3 ± 85.6 | -5.0 (118.3 to 108.2) | 0.93 |

Values are presented as *n*, mean ± standard deviation (SD) or median with range. # Forty-nine incident patients were included before start of medical treatment. *95% CI*: 95% confidence intervals; *IPF*: Idiopathic pulmonary fibrosis; *FVC*: Forced vital capacity; *DLCO*: diffusing capacity of the lung for carbon monoxide; *6MWT*: 6-minute walk test. The same patients were included in a validation study of the King’s Brief Interstitial Lung Disease questionnaire (manuscript submitted)

14 days

|  | **Responders**  **(*n* = 134)** | **Non-responders**  **(*n* = 16)** | **Difference (95% CI)** | **p-value** |
| --- | --- | --- | --- | --- |
| **Gender** |  | | | |
| Male, *n* | 109 | 13 |  | 1.00 |
| Female, *n* | 25 | 3 |  |  |
| **Age,** years, mean ± SD | 72.6 ± 6.1 | 75.0 ± 7.2 | -2.4 (-5.7 to 0.8) | 0.14 |
| **Time since diagnosis**, years, median (range) | 0.5 (0.0 to 9.3) | 0.4 (0.0 to 3.3) |  | 0.45 |
| **Smoking status** |  | | | |
| Current, *n* | 6 | 3 |  | 0.03* |
| Former, *n* | 94 | 7 |  |  |
| Never, *n* | 34 | 6 |  |  |
| **Long-term oxygen therapy** |  | | | |
| No, *n* | 116 | 15 |  | 0.70 |
| Yes, *n* | 18 | 1 |  |  |
| **Medical treatment for IPF#** |  | | | |
| No, *n* | 59 | 6 |  | 0.79 |
| Yes, *n* | 75 | 10 |  |  |
| **FVC**, % predicted, mean ± SD | 87.1 ± 23.2 | 88.4 ± 22.6 | -1.3 (-13.4 to 10.8) | 0.83 |
| **DLCO**, % predicted, mean ± SD | 47.9 ± 14.0 | 52.9 ± 14.3 | 5.0 (-12.3 to 2.4) | 0.18 |
| **6MWD**, m, mean ± SD | 457.5 ± 107.5 | 394.8 ± 137.9 | 62.7 (4.4 to 121.1) | 0.04* |

Values are presented as *n*, mean ± standard deviation (SD) or median with range. * p<0.05. # Foesrty-nine incident patients were included before start of medical treatment. *95% CI*: 95% confidence intervals; *IPF*: Idiopathic pulmonary fibrosis; *FVC*: Forced vital capacity; *DLCO*: diffusing capacity of the lung for carbon monoxide; *6MWD*: distance walked during the 6-minute walk test
