# Supplementary material for: Validation of the IPF-specific version of St. George’s Respiratory Questionnaire
Source: Respir Res. 2019 Aug 28;20:199. doi: 10.1186/s12931-019-1169-9 (PMC6714302; doi:10.1186/s12931-019-1169-9)
Supplement: Supplementary file 6 — Known groups validity. (DOCX 15 kb) [file 12931_2019_1169_MOESM6_ESM.docx]

**Additional file 6: Known groups validity**

| **Variable** | ***n*** | **Mean SGRQ-I total score (SD)** | | **Mean difference (95% CI)** | **p-value** | **ES** |
| --- | --- | --- | --- | --- | --- | --- |
| **FVC** |  |  |  |  |  |  |
| Lower quartile | 37 | 55.3 (19.9) | | 18.1  (9.0 to 27.2) | 0.0002 | 0.18 |
| Higher quartile | 39 | 37.2 (19.9) | |  |  |  |
| **DLCO** |  |  |  |  |  |  |
| Lower quartile | 35 | 55.8 (19.4) | | 26.6  (17.2 to 36.0) | <0.0001 | 0.31 |
| Higher quartile | 37 | 29.2 (20.5) | |  |  |  |
| **LTOT** |  |  |  |  |  |  |
| No LTOT | 129 | 40.1 (22.0) | | 20.9* | 0.0001 | 0.10 |
| Receiving LTOT | 19 | 61.1 (14.5) | |  |  |  |
| **GAP index** |  |  |  |  |  |  |
| 1 | 56 | 34.2 (22.4) | |  | <0.0001 | 0.14 |
| 2 | 75 | 45.2 (19.8) | |  |  |  |
| 3 | 17 | 60.6 (20.4) | |  |  |  |

*: No 95% CI due to the use of a non-parametric test. *SGRQ-I*: IPF-specific version of the St. Georges Respiratory Questionnaire. *FVC*: Forced vital capacity. *DLCO*: diffusing capacity of the lung for carbon monoxide. *LTOT*: Long-term oxygen therapy; *GAP index*: Gender, age, physiology index. *95% CI*: 95% confidence intervals. *ES*: Effect size (partial η^2^).
